# What Influences the Financial Literacy of Young Adults? A Combined Analysis of Socio-Demographic Characteristics and Delay of Gratification

**DOI:** 10.3389/fpsyg.2021.663254

**Published:** 2021-12-02

**Authors:** Christin Siegfried, Eveline Wuttke

**Affiliations:** Faculty of Economics and Business, Business Education, Goethe-University Frankfurt, Frankfurt, Germany

**Keywords:** financial literacy, financial well-being, moderator, delay of gratification, structural equation modeling, influencing factors

## Abstract

The current economic landscape is complex and globalized, and it imposes on individuals the responsibility for their own financial security. This situation has been intensified by the COVID-19 crisis, since short-time work and layoffs significantly limit the availability of financial resources for individuals. Due to the long duration of the lockdown, these challenges will have a long-term impact and affect the financial well-being of many citizens. Moreover, it can be assumed that the consequences of this crisis will once again particularly affect groups of people who have already frequently been identified as having low financial literacy. Financial literacy is therefore an important target for educational measures and interventions. However, it cannot be considered in isolation but must take into account the many potential factors that influence financial literacy alone or in combination. These include personality traits and socio-demographic factors as well as the (in)ability to defer gratification. Against this background, individualized support offers can be made. With this in mind, in the first step of this study, we analyze the complex interaction of personality traits, socio-demographic factors, the (in-)ability to delay gratification, and financial literacy. In the second step, we differentiate the identified effects regarding different groups to identify moderating effects, which, in turn, allow conclusions to be drawn about the need for individualized interventions. The results show that gender and educational background moderate the effects occurring between self-reported financial literacy, financial learning opportunities, delay of gratification, and financial literacy.

## Introduction

In recent years, citizens of most industrialized countries have experienced an increasing degree of complexity and uncertainty in social and economic contexts. This has been intensified by the COVID-19 crisis (Van Dalen and Henkens, 2020). Short-time work and layoffs significantly limit the availability of financial resources for individuals. Due to the long duration of the lockdown, this will have a long-term impact and affect the financial well-being of many citizens. It can be assumed that the consequences of this crisis will once again particularly affect groups of people who have already frequently been identified as having low financial literacy, such as women, people with a migration background, and those with an educationally distant background, since these people are often also employed in sectors that have been particularly affected by the crisis.

Generally, a shifting balance between financial buffers and efficiency and a newly developing understanding of personal responsibility, as indicated not least by the increasing withdrawal of states from support systems, such as pension and health insurance, increase citizens’ requirements to make financially sound decisions (Van Dalen and Henkens, 2020). However, to make financial decisions, individuals need not only to have financial literacy but also to fulfill their social role as responsible citizens (Aprea et al., 2016) and to reach a desired state of financial well-being. Following Brüggen et al. (2017), we define financial well-being as the perception of being able to sustain the current and anticipated desired living standards and financial freedom. To be able to achieve this goal, finance-related knowledge, skills, and attitudes – usually termed financial literacy – are crucial and are increasingly attracting the attention of politicians and scientists. A high level of financial literacy is considered as conditioning sensible financial decisions (e.g., Braunstein and Welch, 2002). If people are financially literate, they are supposed to be able to plan and control their personal financial matters, to avoid over-indebtedness, and to provide for their old age by securing their personal financial prosperity (e.g., Lusardi and Mitchell, 2014). Besides being influential at the micro level, financial literacy is considered to be important when it comes to macro-level concerns, like financial stability, as, for example, the experience of the 2008 subprime crisis suggests (e.g., Mitchell and Lusardi, 2015).

If we consider the numerous national and international studies and surveys published in recent years (e.g., Allianz, 2017; OECD, 2017), however, it becomes clear that one must rather assume financial illiteracy of the population of many nations. Studies also show that some groups tend to perform particularly poorly. This is primarily the case for women as well as for persons with a migrant background and/or low levels of education (e.g., Bucher-Koenen et al., 2017; Happ and Förster, 2019), although the recent study by Wuttke et al. (2020) reports different results. These results are usually attributed to different individual dispositions, such as an interest in financial issues (e.g., Brown and Graf, 2013; Lührmann et al., 2013), or different socialization patterns and learning opportunities (e.g., Rinaldi, 2017; Rudeloff, 2019).

In these studies, two aspects are often neglected:

1. One factor can be described as impulse problems, which might play a decisive role when making financial decisions. Studies using self-reports reveal that participants with high levels of debt often report a lack of self-control, the inability to delay gratification, and the pleasure experienced when spending (O’Guinn and Faber, 1989; Lunt and Livingstone, 1991; Livingstone and Lunt, 1992). Furthermore, the inability to delay gratification is a significant predictor of debt because it often results in impulse buying (e.g., Norwilitis et al., 2006). Although impulse buying may have a positive short-term impact on a person’s emotional state, it does not contribute to financial well-being in the long term. Therefore, consideration of the ability to delay gratification is crucial when analyzing and supporting financial literacy.
2. A further problem is that many studies focus on an isolated analysis of variables influencing financial literacy. Predominantly, the effect of specific socio-demographic factors, such as gender, age, educational background, or migration background, is taken into consideration (e.g., Lusardi and Mitchell, 2014; Ergün, 2017; Gramațki, 2017; Strömbäck et al., 2017; Happ and Förster, 2019; Rudeloff et al., 2019). Studies dealing with delay of gratification is also mainly interested in the influence of (isolated) socio-demographic factors on this variable (Benjamin et al., 2020), and only a few studies combine socio-demographic factors, delay of gratification, and financial literacy in their analyses (Hastings and Mitchell, 2020). Such an isolated consideration of the cause-and-effect relationship often leads to the conclusion that consumers who have trouble with financial decisions just lack an understanding of simple economic concepts and cannot carry out computations or assumes that the inability to delay gratification or a present bias leads people to choose immediate gratification and make suboptimal financial decisions. In our study, we follow studies that use a combined approach since it can be expected that personality traits, socio-demographic factors, and the (in-)ability to delay gratification interact in a complex way and influence financial literacy, financial decisions, and, in the long run, financial well-being. The latter is achieved by a healthy spending and savings balance, which is crucial for sustaining long-term financial and personal well-being (Van Praag et al., 2003). Achieving this, however, requires financial literacy and self-control.

To pursue the investigation of the complex structure of influencing factors, we proceed as follows. In section “Definition of Financial Literacy,” we define the construct of financial literacy. In section “Factors Influencing Financial Decisions,” we describe the state of research on financial literacy and delay of gratification and focus especially on the factors influencing financial literacy, such as gender, migration background, education, and delay of gratification. We also take moderating effects on financial literacy into account. We then outline the research questions and the study design (section “Research Questions, Study Design and Instruments”) and present the results of the study (section “Results”). These results, as well as the limitations of the study, are then discussed, and conclusions are drawn with regard to further steps (section “Discussion”).

## Definition of Financial Literacy

In our study, we adopt a holistic, competence-oriented view of financial literacy, defined as the potential that enables a person to plan, execute, and control financial decisions effectively. This potential is based on the availability of individual dispositions (knowledge and skills, motivations and interests, attitudes, and values) and contingent on situational characteristics (e.g., Weinert, 2001; Aprea et al., 2016). We differentiate two dimensions of the construct. The first dimension refers to the contextual perspective from which financial literacy is considered, and it comprises the “individual versus systemic” categories. The characteristic “individual” focuses on the individual as a consumer making financial decisions in the personal and market environments, whereas the “systemic orientation” characteristic subsumes issues of the larger economic and social context as well as the economic and political framework conditions. The second dimension represents the “personal resources,” which can be categorized into “cognitive” and “non-cognitive.” The “cognitive” category refers mainly to knowledge, skills, and abilities, while “non-cognitive” dispositions imply emotional, motivational, and volitional aspects, as well as social values and norms, which can also be understood as personal traits and characteristics.^1^ The combination of these dimensions leads to four competence areas: the individual cognitive, individual non-cognitive, systemic cognitive, and systemic non-cognitive areas (introduced by Aprea et al., 2016; see Table 1). The test used in our study follows this logic (see section “Factors Influencing Financial Decisions”).

**Table 1:** Facets of financial literacy (see also Leumann et al., 2016).

	Individual Perspective	Systemic Perspective
	“Manager” of personal finance Ability for effective and efficient financial decisions Ability to reflect critically upon financial related decisions and to use them responsibly	Mature economic citizen in financial issues Ability to understand and participate actively in a democratic economic and financial system
Cognitive resources (knowledge, skills, abilities)	Individual cognitive	Systemic cognitive
Non-cognitive resources (interests, attitudes, values)	Individual non-cognitive	Systemic non-cognitive

These resources are crucial to sustain the current and attain the aspired living standard as well as financial freedom as central facets of financial well-being.

## Factors Influencing Financial Decisions

### Socio-Demographic Factors

Different definitions of the construct financial literacy are used across studies, the operationalization of socio-demographic factors (e.g., migration background or educational background) varies, and the methods applied in studies are quite different. Furthermore, studies are from different countries. Therefore, their results are not quite comparable. Nonetheless, the results of many studies point to the fact that gender, migration background, educational background, and opportunities to learn are important for the development of financial literacy and can therefore be identified as central influencing factors. Moreover, the presence of some of these factors, such as gender or educational background, can moderate (i.e., enhance or weaken) the relationship between other influencing factors and financial literacy (e.g., Perry and Morris, 2005; Lusardi, 2011; Greimel-Fuhrmann and Silgoner, 2018; Longobardi et al., 2018; Rudeloff et al., 2019).

#### Gender

Most studies indicate that men perform better than women in financial literacy tests (e.g., Lusardi and Mitchell, 2014; Schürkmann and Schuhen, 2014; Ergün, 2017; Gramațki, 2017; OECD, 2017; Strömbäck et al., 2017; Förster et al., 2018). Only a few studies show no gender differences, and this is only true for some facets of financial literacy (e.g., Hill and Asarta, 2016; Strömbäck et al., 2017; Greimel-Fuhrmann and Silgoner, 2018; OECD, 2018; Rudeloff et al., 2019; Santini et al., 2019; Wuttke et al., 2020). One explanation for the often-found gender gap is that men are more likely to be part of groups with finance-related interests and therefore achieve higher levels of financial literacy (e.g., Lusardi and Mitchell, 2011). In line with this assumption, some studies indicate that women are less interested in financial issues than men are (Lührmann et al., 2013) and are therefore less motivated to learn about financial contents. Thus, although learning opportunities in financial topics might exist in a similar way for women and men, they use them differently (Goldsmith et al., 1997). On the other hand, a meta-study by Kaiser and Menkhoff (2017) points out that gender has no influence on the effect of financial education. Another explanation in this context can be that woman use different sources of information from men; for example, the financial literacy of women is positively and significantly affected by having many books at home, whereas such a relationship can hardly be found for men (Longobardi et al., 2018). Other studies take traditional roles and traditional divisions of tasks within households into account. In this context, it is assumed that it is mainly men who make financial decisions and that women therefore only build up financial knowledge when it is necessary (Greimel-Fuhrmann and Silgoner, 2018). Findings assuming that the gender gap is larger for younger test participants than for older ones (Greimel-Fuhrmann and Silgoner, 2018) seem to be in line with these arguments (see also Hsu, 2016).

#### Migration Background

A further negative influence on financial literacy can arise if the test takers have a migration background (Gramațki, 2017; Happ et al., 2018; Happ and Förster, 2019; Rudeloff et al., 2019). This effect is explained by the fact that immigrants often have a poorer economic background since their parents mainly work in lower-skilled jobs and therefore have a lower income and do not speak the test language at home (Perry and Morris, 2005). Kaiser and Menkhoff (2017) show, in their meta-study, that people with low incomes are less able to take advantage of learning opportunities in finance-related topics than people with higher incomes. Studies that take into account the generation in which the migration has taken place find that the strongest negative effect is recorded for the first-generation immigrants. The effect decreases continuously with the second and third generations (Gramațki, 2017). If a distinction is also made regarding whether the migration background is merely due to the country of origin or whether the spoken language at home is different, the results indicate that native speakers in particular have an advantage in financial literacy (Brown and Graf, 2013; Cameron et al., 2014; Happ and Förster, 2019).

#### Age

A closer look at the influence of age shows that an effect of age is often reported (Gramațki, 2017; Kaiser and Menkhoff, 2017; Strömbäck et al., 2017). However, Lusardi and Mitchell (2014) point out that this applies to different age groups in different ways. According to their studies, financial literacy increases with age (see also Happ and Förster, 2019) but again turns out to be quite low for participants aged 50 and older.

#### Educational Background

Participants with a higher level of education, such as a master’s or PhD degree, appear to have higher financial literacy (e.g., Lusardi and Mitchell, 2014; Ergün, 2017; Gramațki, 2017; Santini et al., 2019). Other studies, which use the number of years of schooling completed, refer to either negative (Kaiser and Menkhoff, 2017; Happ et al., 2018) or positive effects (Gramațki, 2017; Strömbäck et al., 2017). Although there are inconsistent results, most studies assume that the higher the level of general education, the higher the chance that test takers will be better able to answer questions in financial literacy tests. However, studies using the educational background not only as a predictor but also as a moderator point to the fact that the comparison of the degree of financial literacy of higher-educated women and men seems to enlarge the gender gap, with an advantage for male participants (Greimel-Fuhrmann and Silgoner, 2018).

#### Learning Opportunities in Financial Literacy

Another predictor of financial literacy is the extent to which learning opportunities in financial education are available. Studies generally point to a positive impact of learning opportunities (Kaiser and Menkhoff, 2017; Rudeloff et al., 2019). On the other hand, the results of some studies indicate that school-based learning opportunities in financial topics only play a marginal role in the development of financial literacy and that informal learning opportunities, such as those created through discussions within families, seem to have a large positive influence (OECD, 2017; Rudeloff, 2019; Rudeloff et al., 2019; Grohs-Müller, 2020). Thus, parents who manage their money well and talk about it are likely to influence their children to behave similarly (Lusardi, 2011). Shim et al. (2010) show that the role of parents’ financial knowledge and behavior is more relevant for young adults than learning opportunities at school. If there is a lack of financial education at home, the influence of other sources of information, such as advertising and peers (Greimel-Fuhrmann, 2018), increases accordingly. However, Rudeloff et al.’s (2019) results show that, in contrast to discussions with parents, discussions with peers do not necessarily have a positive impact on financial literacy. In light of the fact that learning opportunities are not always a particularly good predictor of financial literacy, it is interesting to note that Lusardi (2011) finds self-assessment of financial knowledge to be a good predictor of actual financial literacy, but this is particularly true for younger respondents, while older respondents often overestimate their financial knowledge.

### Delay of Gratification and Its Influencing Factors

The ability to delay gratification (also: delay of need and delay of gratification; Mischel and Ebbesen, 1970; Mischel et al., 1989; Forstmeier et al., 2011) proves to be a reliable predictor of a successful life in many studies. Self-imposed delay of gratification is regarded as an early indicator of a stable personality trait and reliably predicts the development of cognitive and social competence of adults (e.g., Mischel et al., 1989). This is a central result of a well-known study in which small children were given the choice of receiving more candy (marshmallows) later or a smaller amount immediately (Mischel et al., 1989).^2^

Reward postponement acts as a control mechanism and regulates impulsive (and often risky) behavior. In the context of financial decisions, this includes, for example, impulsive and/or status purchases but also more far-reaching financially risky behavior, such as the choice of financial investments (Legge and Heynes, 2009). Persons with a well-developed ability to delay rewards (i.e., with a high degree of self-control) generally save more money (e.g., Baumeister, 2002) and make fewer impulse purchases (Strayhorn, 2002). They are able to weigh long-term goals against short-term desires. This balancing is one of the most difficult decision-making tasks since temptations from the immediate environment of the persons concerned often conflict with the requirements of long-term plans (delay discounting; Hirsh et al., 2008). Decisions made in favor of a smaller, immediate reward over a later, larger reward are an example of a low capacity for reward postponement (e.g., Mischel et al., 1989).

From a pedagogical point of view and considering long-term financial well-being, it is of particular interest to identify this personality trait, especially against the background of an increasing debt ratio. Although it is generally assumed that personality traits are quite resistant to change, a review of longitudinal studies (Roberts and Del Vecchio, 2000) shows that the ability to postpone rewards can be influenced and therefore changed. Thus, different studies focus on the identification of contextual factors (attitudes of the family of origin toward money, saving behavior, and financial knowledge) that can play a promoting or a hindering role in the ability to postpone rewards. Some studies indicate that women have a stronger ability to plan, execute, and especially exert self-control over their financial behavior (Benjamin et al., 2020; not in Reyna and Wilhelms, 2016). These abilities are displayed in the variable delay of gratification and seem to play a key role in converting knowledge into responsible financial behavior (Silverman, 2003; Benjamin et al., 2020). In relation to financial decisions, Johnson and Staten (2010) report that individuals who are more impatient and less risk averse tend to reveal riskier money management and riskier borrowing behavior. Moreover, results show that the delay of gratification is positively correlated with financial literacy (Reyna and Wilhelms, 2016). Participants with a greater ability to delay gratification are less likely to pay only the minimum credit card bill but more likely to pay in full, are less likely to have an overdrawn bank account, save money more frequently, have less credit card debt, allocate less money to spending immediately and more to savings, and are more satisfied with their life as a whole. Taking all these results into account, a high degree of delay of gratification seems to be central, especially for topics that take savings into account (Strömbäck et al., 2017).

Regarding the presented studies and their results, it has to be mentioned that delay of gratification is seen as being influenced by financial literacy (in the form of financial knowledge) and that delay of gratification, in turn, influences financial behavior (the more people know about finance, the more they are able to delay gratification and the wiser their financial behavior is). Since we use a situational judgment test (SJT) in our study, we assume that it is close to measuring financial behavior (Kahmann, 2014). We therefore assume that financial literacy influences the ability to delay gratification and that this, in turn, influences financial behavior.

The studies outlined so far show that, although the findings are not unambiguous, the variables age, gender, migration background, educational background, previous learning opportunities, and delay of gratification can be regarded as having an influence on financial literacy and behavior. For this reason, we include these variables in our study. The findings presented so far mainly come from investigations of correlations between financial literacy on the one side and socio-demographic factors and delay of gratification on the other side. There are some findings that also show an influence of individual characteristics on the delay of gratification.

Based on the studies so far, the relationships between the variables are illustrated graphically. If the previous results are inconclusive, possible relationships are represented with a dashed arrow; if there are clear findings to date, positive relationships are represented with a grey arrow and negative ones with a black arrow.

## Research Questions, Study Design, and Instruments

### Research Questions and Methodological Approach

In our study, we choose an integrated approach to the relationship between delay of gratification and financial literacy, considering the other influencing factors.

This leads to the following hypotheses and research questions:

Hypothesis 1: Gender and opportunities to learn have a positive influence on gratification delay.

Hypothesis 2: Opportunities to learn, self-reported financial literacy, and delay of gratification have a positive influence and migration background a negative influence on financial literacy.

#### Research Question 1

To what extent can socio-demographic characteristics (age, gender, educational background, migration background, and opportunities to learn about finance) be modeled as influencing factors on delay of gratification and financial literacy?

Moreover, the results of the presented studies indicate that socio-demographic characteristics are not only an influencing factor on financial literacy and delay of gratification. Some of them, especially gender and educational level, seem to moderate the relationship between (other) socio-demographic factors and financial literacy and delay of gratification. Therefore, it is worthwhile making a distinction between men and women and between people with different educational backgrounds in our model. Against the background of the current debate on the promotion of financial literacy among young adults and the nearly constant effect regarding gender and educational background on financial literacy, a detailed investigation of this effect would be particularly interesting. This leads to hypotheses 3 and 4 and research question 2:

Hypothesis 3: Gender moderates the relationship between age and opportunities to learn and financial literacy.

Hypothesis 4: Educational background moderates the relationship between gender and financial literacy.

#### Research Question 2

To what extent do gender and educational background change the influence of the remaining characteristics on financial literacy?

**Figure 1:**
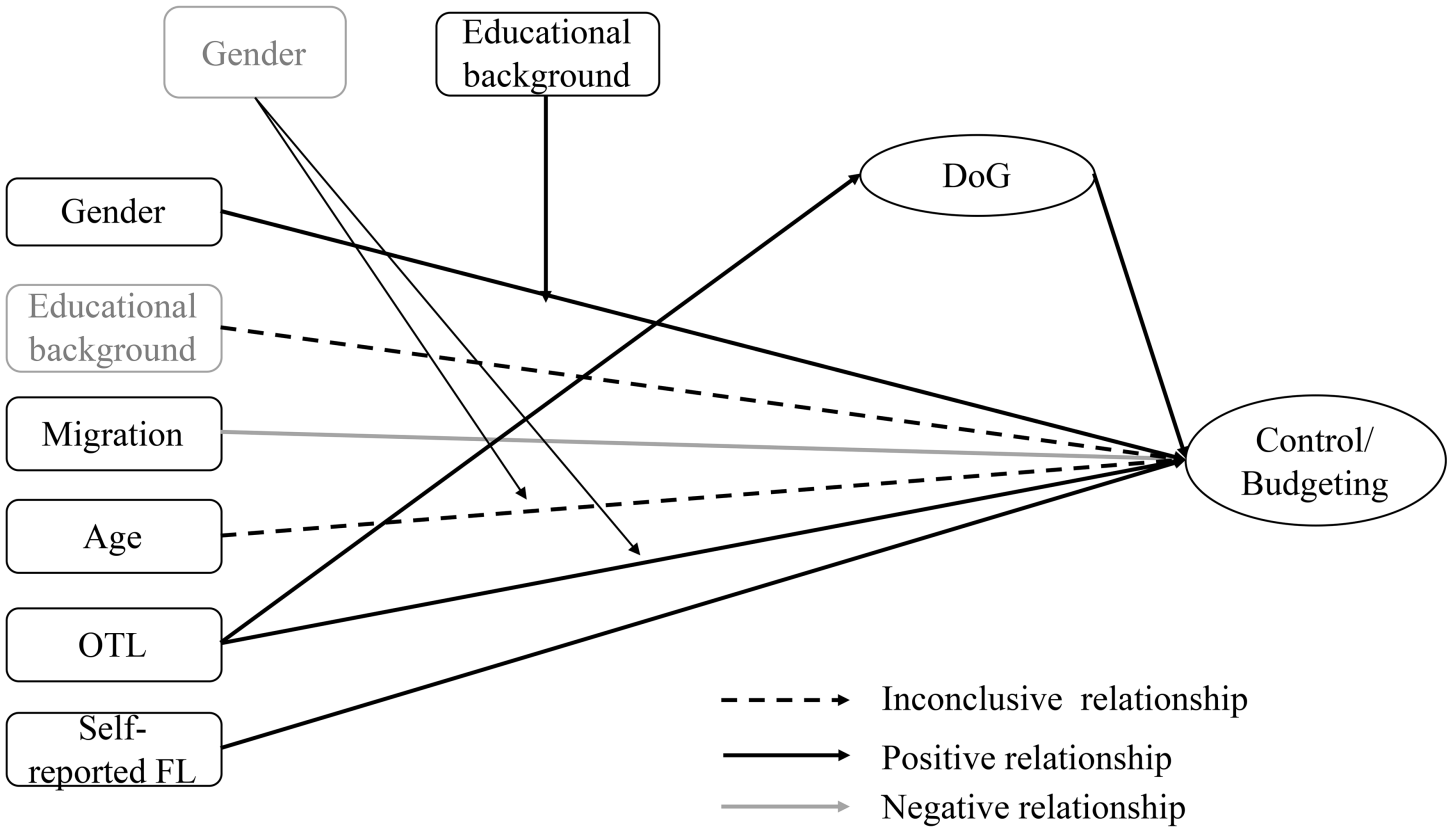
Summary of the findings from previous studies regarding the relationship between individual factors, delay of gratification (DoG), and financial literacy (FL).

**Figure 2:**
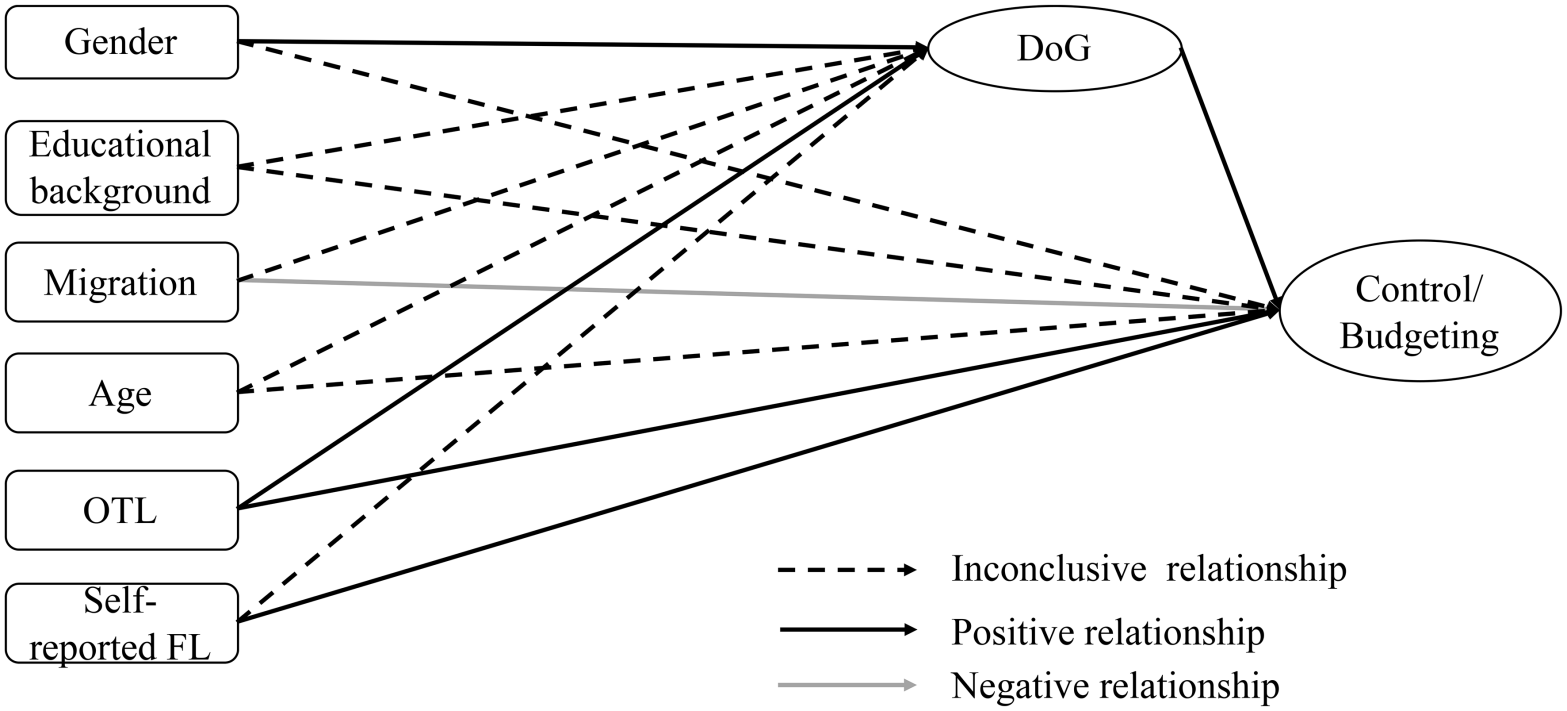
Tested relationships between individual factors, delay of gratification (DoG), and financial literacy (FL).

To answer these questions, two types of analyses will be carried out:

Structural equation modeling will be conducted to investigate the influence of the individual characteristics and the delay of gratification as independent variables on financial literacy as the dependent variable (see Figures 1, 2).

**Figure 3:**
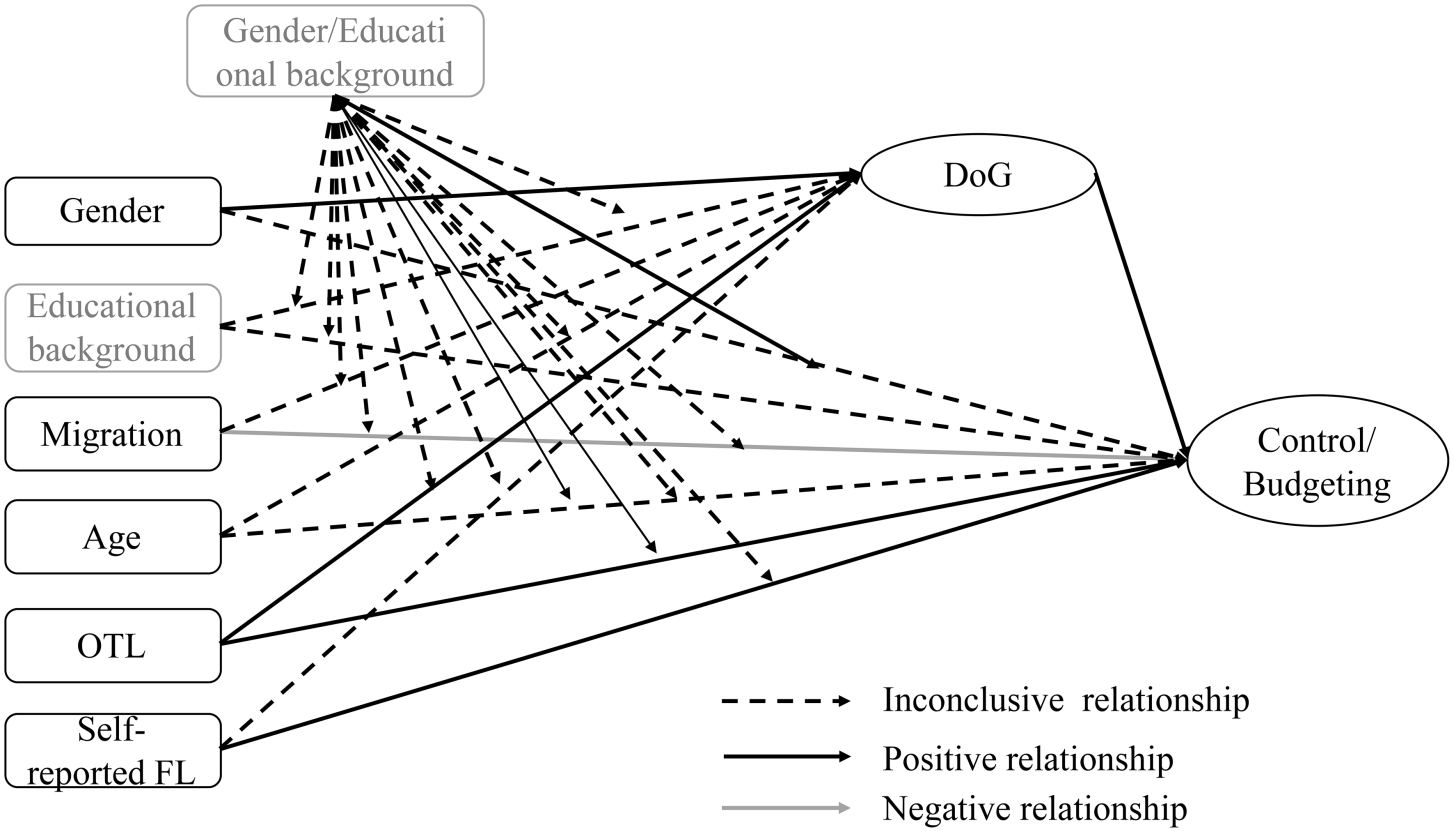
Tested moderator effects on the relationship between individual factors, delay of gratification (DoG), and financial literacy (FL).

To answer the second question, another analysis is required. For this purpose, gender and educational background are defined as a group variable in the structural equation model. In a multi-group analysis, it is thus possible to identify the extent to which the influence of individual characteristics on financial literacy changes in the different groups (see Figure 3) (male/female; academic background/vocational background; see also chapter 4.3).

### Instrument

To measure financial literacy, we use an SJT, which is based on the competence-oriented approach to financial literacy defined above. This approach differentiates between different dimensions and facets of the construct, such as “saving money and building assets,” “borrowing money,” and “comparing and contracting insurance.” In this paper, we focus on the competence facet “planning and managing financial matters of everyday life” (for details of the basic assumptions and elaborations of the competence-oriented approach, *cf.* Aprea and Wuttke, 2016 as well as Leumann et al., 2016). The test for this facet consists of 11 situations with a total of 22 items developed in a previous study (Wuttke and Aprea, 2018). It comprises three factors that explain 39% of the variance:

– Overview/control of one’s own financial situation (9 items, max. 36 points, *α*=0.754)
– Budgeting (6 items, max. 24 points, *α*=0.573)
– Sensible handling of money (7 items, max. 28 points, *α*=0.691).

Furthermore, we collected demographic data, such as age, gender, migration background, family background, and educational background. For the variable delay of gratification, we administer the buying behavior and delay of gratification scale by Ray and Najman (1986). An example item is “When someone gives me money, I prefer to spend it right away.” The data collection took place in 2016/2017.

### Sample

Tests with many missing items are removed from the sample. The resulting sample is *N*=206 (see Table 2).

**Table 2:** Overview of the sample.

		**N**
Type of school or university	University, Business/Business Education	28
	University, Educational Science	32
	University, Study program not specified	45
	Full-time vocational schools, dual vocational education (drafting, carpentry, specialty in removal services)	101
Age	16–25years, mean: 20.6years	
Gender	108=female, 93=male, 5=no answer	
Native language German	German as mother tongue of parents: 62%	
			206

A total of 149 participants in the sample have a migration background. We operationalize the migration background *via* the mother tongue of the participants. Regarding the educational background, the sample can be divided into two groups: Participants have either an academic background (university students, *N*=105) or a vocational background (students in full-time vocational schools or in dual vocational education, *N*=101). Regarding previous opportunities to learn (OTL) about finance-related topics, we refer to formal learning opportunities. A distinction is made between persons who have had such opportunities during general and/or vocational education and training and persons who have not yet had access to systematic OTL in financial topics during their school career (OTL in finance-related topics, *N*=98; no OTL in finance-related topics, *N*=102; missing values=6). For the assessment of financial literacy, the participants were asked how educated they felt about financial topics (four-level Likert scale; 1=educated to 4=uneducated).

## Results

### Factors Influencing Financial Literacy (Hypotheses 1 and 2 and Research Question 1)

With reference to the previous analyses by Wuttke et al. (2020), who show that the third facet – “sensitive handling of money” – does not achieve partial strict measurement invariance, only the first two facets of the test are considered in the following. To answer research question 1, the relationship between the independent variables, namely, age, gender, educational background, migration background, opportunities to learn about finance, and delay of gratification, and the dependent variables, specifically the facets *control* and *budgeting*, is investigated using a structural equation model. The two facets of financial literacy, *control* and *budgeting*, together with *delay of gratification*, are modeled latently. Gender, age, domain-related prior knowledge, migration background, educational background, and degree of self-reported financial literacy are included in the model as exogenous, manifest variables. For this, the AMOS software (Arbuckle, 2016) is used. Due to the presence of missing values, the full information maximum likelihood method (FIML method) is used for the model estimation. For the implementation of this method, normal multivariate distribution is usually required (Weiber and Mühlhaus, 2014). Multivariate normal distribution on the basis of the Mardia coefficient cannot be calculated due to missing values, even though they are replaced directly in the model during parameter estimation using the FIML method. However, following Arzheimer (2015), the maximum likelihood estimation algorithm is nevertheless used as the biases are classified as small in the case of violation of the multivariate normal distribution.

The model quality criteria indicate a good fit of the empirical data to the theoretical model for both control (*χ*^2^=119,775, df=91, *p*=0.02, CFI=0.94, RMSEA=0.039, PCLOSE=0.826) and budgeting (*χ*^2^=88,624, df=62, p=0.02, CFI=0.94, RMSEA=0.046, PCLOSE=0.613) (Hu and Bentler, 1999). The standardized regression weights are reported in Table 3.

**Table 3:** Standardized regression weights for the structural equation model for the two facets of financial literacy, control and budgeting.

	Control (*R*^2^=0.40)			Delay of gratification (*R*^2^=0.08)			Budgeting (*R*^2^=0.28)			Delay of gratification (*R*^2^=0.08)		
	B	SE B	*β*	B	SE B	*β*	B	SE B	*β*	B	SE B	*β*
Gender (0=male, 1=female)	0.24	0.08	0.25^**^	1.07	0.92	0.09	0.04	0.10	0.04	1.02	0.92	0.08
Educational background (0=vocational, 1=academic)	−0.16	0.09	−0.17^*^	2.12	1.01	0.17^*^	0.18	0.11	0.14	2.18	1.01	0.18^*^
Migration background (0=without, 1=with)	−0.01	0.08	−0.01	1.47	1.02	0.11	−0.06	0.11	−0.04	1.46	1.03	0.11
Age	0.00	0.01	0.02	−0.08	0.13	−0.04	−0.03	0.02	−0.17^*^	−0.08	0.13	−0.04
OTL in finance (0=no, 1=yes)	−0.06	0.07	−0.07	0.24	0.84	0.02	0.05	0.09	0.04	0.23	0.84	0.02
Self-reported FL (1=low-4=high)	0.04	0.05	0.06	−0.61	0.64	−0.07	−0.13	0.07	−0.14	−0.59	0.64	−0.07
Delay of gratification	0.05	0.01	0.586^**^				0.04	0.01	0.41^**^			
Modelfit	*χ*^2^ =119,775, df=91,*p* =0.02, CFI=0.94, RMSEA=0.039, PCLOSE=0.826						*χ*^2^ =88,624, df=62, p=0.02, CFI=0.94, RMSEA=0.046, PCLOSE=0.613					

* *p*<0.05,

** *p*<0.01.

Gender (*β*=0.25), educational background (*β*=−0.17), and delay of gratification (*β*=0.59) are significant predictors of the control facet, whereas age (*β*=−0.17) and delay of gratification (*β*=0.41) are significant predictors of the budgeting facet. Thus, it can be shown that neither OTL about finance nor migration background play a decisive role. The best predictor for the two facets of financial literacy is the ability to delay gratification. The explained variance is 40% for control and 28% for budgeting. For delay of gratification, only the educational background (in the model for control: *β*=0.18; for budgeting: *β*=0.18) is a significant predictor and, together with the other predictors, explains 8% of the variance. Thus, hypotheses 1 cannot be confirmed and hypothesis 2 only partially, since at least delay of gratification has a positive influence on financial literacy.

### Moderating Effect of Gender (Hypothesis 3 and Research Question 2)

To test the hypothesis 3 and research question 2 of a moderating effect of gender, a multi-group analysis is conducted. For this purpose, the model presented earlier is used and the relationships between socio-demographic characteristics, delay of gratification, and financial literacy are analyzed separately for women and men. Thus, the variable gender serves as a group variable to compare the effects between the dependent and the independent variables. The fit values of the theoretical two-group models to explain the *budgeting* (F1) and *control* (F2) facets show a good fit with the empirical data (F1: *χ*^2^=206,919, df=174, *p*=0.045, CFI=0.91, RMSEA=0.031, PCLOSE=0.984; F2: *χ*^2^=164,846, df=116, *p*=0.002, CFI=0.89, RMSEA=0.046, PCLOSE=0.648) (Hu and Bentler, 1999). To determine which regression weights differ significantly between female and male test takers, different models are established, each restricting a single regression coefficient to be equal for both men and women. The results show that the model fit is significantly worse if no distinction is made between the regression coefficients of (1) self-reported financial literacy (model control: df=1, CMIN=5,04, *p*=0.025; model budgeting: df=1, CMIN=7,111, *p*=0.029) on delay of gratification and (2) OTL about finance (df=1, CMIN=6,171, *p*=0.046) on budgeting between the genders. No regression weight causes any significant model deterioration when restricted to equality.

For the comparison of the two groups, all the regression weights are reported in Table 4 as both non-standardized and standardized values and their standard deviation.

**Table 4:** Standardized regression weights for the structural equation model for the moderating effect of gender.

**Female**	**Control (*R*** ^**2**^ **=0.46)**			**Delay of gratification (*R*** ^**2**^ **=0.10)**			**Budgeting (*R*** ^**2**^ **=0.34)**			**Delay of gratification (*R*** ^**2**^ **=0.08)**			B	SE B	*β*	B	SE B	*β*	B	SE B	*β*	B	SE B	*β*
Educational background (0=vocational, 1=academic)	−0.19	0.09	−0.24^*^	1.01	1.19	0.09	0.16	0.13	0.13	0.20	0.97	0.09
Migration background (0=without, 1=with)	0.09	0.10	0.10	−2.55	1.34	−0.20^*^	−0.05	0.15	−0.04	−2.55	1.34	−0.20^*^
Age	0.004	0.011	−0.03	0.086	0.16	0.05	−0.041	0.017	−0.24^*^	0.09	0.16	0.05
OLT in finance (0=no, 1=yes)	0.002	0.069	0.003	0.20	0.97	0.02	0.232	0.109	0.22^*^	0.20	0.97	0.02
Self-reported FL (1=low-4=high)	−0.01	0.05	−0.02	−0.77	0.73	−0.10	0.22	0.08	0.28^**^	−0.77	0.73	−0.10
Delay of gratification	0.05	0.01	0.68^**^				0.04	0.01	0.42^**^	0.20	0.97	0.02
**Male**	**Control (*R*** ^**2**^ **=0.33)**			**Delay of gratification (*R*** ^**2**^ **=0.10)**			**Budgeting (*R*** ^**2**^ **=0.24)**			**Delay of gratification (*R*** ^**2**^ **=0.10)**			B	SE B	*β*	B	SE B	*β*	B	SE B	*β*	B	SE B	*β*
Educational background (0=vocational, 1=academic)	−0.09	0.12	−0.09	3.21	1.67	0.21^*^	0.13	0.17	0.09	3.25	1.66	0.21^*^
Migration background (0=without, 1=with)	−0.01	0.11	−0.01	−0.66	1.58	−0.05	−0.12	0.16	−0.08	−0.62	1.58	−0.04
Age	0.00	0.01	0.03	−0.18	0.21	−0.09	−0.03	0.02	−0.14	−0.18	0.21	−0.09
OLT in finance (0=no, 1=yes)	−0.16	0.10	−0.17	−0.08	1.41	−0.01	−0.12	0.14	−0.09	−0.10	1.41	−0.01
Self-reported FL (1=low-4=high)	−0.11	0.07	−0.16	2.15	1.07	0.21^*^	0.04	0.10	0.04	2.14	1.07	0.20^*^
Delay of gratification	0.04	0.01	0.57^**^				0.04	0.01	0.42^**^			
Modelfit	*χ*^2^ =206,919, df=174, *p* =0.045, CFI =0.91, RMSEA=0.031, PCLOSE=0.984;						*χ*^2^ =164,846, df=116, *p* =0.002, CFI =0.89, RMSEA=0.046, PCLOSE=0.648					

* *p*<0.05,

** *p*<0.01.

The regression weights show that, for female in contrast to male respondents, the migration background (*β*=−0.20) influences the degree of delay of gratification significantly, but the migration background has no significant influence on the two facets control and budgeting for both genders. This means that female test persons without a migration background have a greater ability to delay gratification than women with a migration background. Other factors influencing delay of gratification cannot be identified for female participants. Interestingly, completely different factors seem to influence male participants’ ability to delay gratification. Educational background (*β*=0.21) and self-reported financial literacy (model control: *β*=0.21; model budgeting: *β*=0.20) have a significant positive effect on male subjects’ ability to delay gratification. This means that males with an academic background and higher self-reported financial literacy can control their finances better than male participants with a vocational background.

Regarding the influencing factors for the two facets of financial literacy, the results demonstrate that, for female participants, an influencing factor for the control facet is the educational background (*β*=−0.24). This means that females with an academic background are less able to control their finances than apprentices in vocational education and training. For the budgeting facet, age (*β*=−0.24), having attended OTL about finance (*β*=0.22), and self-reported financial literacy (*β*=0.28) are further influencing factors for women. In contrast, the highly significant influence of the delay of gratification on both facets (control female: *β*=0.68, control male: *β*=0.57; budgeting female: *β*=0.42, budgeting male: *β*=0.42) exists independently of gender. For men, no more significant influencing factors (apart from delay of gratification) can be identified. However, as already described, only the effects of self-reported financial literacy on delay of gratification and OTL about finance on the budgeting facet differ significantly between the genders. This means that at least hypothesis 3 can be partially confirmed.

### Moderating Effect of Educational Background (Hypothesis 4 and Research Question 2)

To test the moderating effect of the educational background (Hypothesis 4 and research question 2), a multi-group analysis is carried out (0=with academic background, 1=with vocational background), as was previously performed for gender. This means that the model already described (research question 1) is used, and these relationships between independent and dependent variables are examined in relation to the educational background. The fit values of the theoretical two-group model show a good fit with the empirical data for the *control* facet (F1: *χ*^2^=206,873, df=177, *p*=0.062, CFI=0.92, RMSEA=0.029, PCLOSE=0.99), whereas the model fit for the *budgeting* facet does not reach an adequate level (*χ*^2^=179,407, df=119, *p*<0.01, CFI=0.84, RMSEA=0.05, PCLOSE=0.49; Hu and Bentler, 1999). Thus, only the results for the first facet (control) are presented.

Comparing different models and restricting a single regression coefficient to be equal for both groups, the model fit is significantly worse if no distinction is made between the regression coefficients of self-reported financial literacy (df=1, CMIN=2,992, *p*=0.084) and the delay of gratification between the groups. All the other regression weights do not cause any significant model deterioration when restricted to equality.

For the comparison of the two groups, all the regression weights are reported in Table 5 as both non-standardized and standardized values and their standard deviation.

**Table 5:** Standardized regression weights for the structural equation model for the moderating effect of educational background.

	Academic						Vocational					
	Control (*R*^2^=0.28)			Delay of gratification (*R*^2^=0.10)			Control (*R*^2^=0.41)			Delay of gratification (*R*^2^=0.07)		
	B	SE B	*β*	B	SE B	*β*	B	SE B	*β*	B	SE B	*β*
Gender (0=male, 1=female)	0.15	0.09	0.16	0.02	1.01	0.002	0.34	0.132	0.27^*^	2.03	1.52	0.13
Migration background (0=without, 1=with)	−0.01	0.15	−0.01	0.74	1.65	0.04	0.01	0.11	0.01	−2.06	1.40	−0.14
Age	−0.003	0.02	−0.07	0.1	0.19	0.05	0.02	0.01	0.11	−0.14	0.19	−0.08
OLT in finance (0=no, 1=yes)	−0.03	0.08	−0.03	0.81	0.92	0.09	−0.12	0.11	−0.10	−0.41	1.43	−0.03
Self-reported FL (1=low-4=high)	0.004	0.06	0.01	−0.52	0.67	−0.08	−0.12	0.08	−0.14	1.71	1.09	0.16
Delay of gratification	0.06	0.01	0.64^**^				0.04	0.01	0.54^**^			
Modelfit	*χ*^2^ =206,873, df=177, *p* =0.062, CFI =0.92, RMSEA=0.029, PCLOSE=0.99											

* *p*<0.05,

** *p*<0.01.

Table 5 shows that, for participants with an academic background but also for apprentices in vocational education and training, none of the measured individual characteristics influences the degree of delay of gratification significantly. However, delay of gratification is a significant influencing factor for the facet control of financial literacy (academic: *β*=0.64; vocational: *β*=0.54) for both groups. Meanwhile, for academic participants, no other influencing factors prove to be significant for the control facet, and gender (*β*=0.24) seems to be an additional relevant influencing factor for apprentices in vocational education and training. This means that only for apprentices can the assumption be made that women perform better than men in this facet of financial literacy. In summary, hypothesis 4 cannot be confirmed.

However, as already described, only the effects of self-reported financial literacy on delay of gratification – even if they are not significant for persons with either an academic or a vocational educational background – differ significantly between the two groups. This is mainly due to the contrasting influence of self-reported financial literacy on delay of gratification. For subjects with an academic background, there is a negative relationship between self-reported financial literacy and delay of gratification, which means that lower self-reported financial literacy is associated with a longer delay of gratification. This relationship between self-reported financial literacy and delay of gratification is positive for participants with a vocational education background.

## Discussion

The aim of this paper was to provide a more detailed insight into the relationship between individual characteristics, delay of gratification, and financial literacy.

Direct influences on financial literacy can be identified by gender (control facet), educational background (control facet), and the ability to delay gratification (both facets). The ability to delay gratification is in turn influenced by the educational background. This makes it clear that delay of gratification is a particularly significant influencing factor on financial literacy. This is not always true for all facets of financial literacy when looking at gender, educational background, and age.

The aim of the article was to investigate these relationships further by analyzing the moderating effects of gender and educational background.

Gender as a moderator:

– While OTL about finance seems especially to enable women to perform better in the financial literacy test, it does not play a significant role for men. This is particularly interesting since it can initially be assumed that similar school careers took place for men and women, and thus, similar learning opportunities were offered (but probably not really used). One explanation is that the learning opportunities on finance-related topics that are currently offered at schools do not really provide the necessary knowledge for reasonable financial behavior (see also Rudeloff, 2019). Since young women obviously use these learning opportunities, it is important to offer them in schools. For young men, it is necessary to consider or analyze why they do not use these learning opportunities successfully and whether they may need another form of learning opportunities (see also Rudeloff et al., 2019). Further qualitative studies, such as interviews, are necessary for this.
– A high degree of (self-reported) financial literacy as well as the educational background seem to be important predictors of delay of gratification among men. Thus, it can be assumed (1) that males assess their financial literacy more realistically than females (because this is the expected relationship: individuals who have higher finance-related knowledge are more likely to delay gratification); and (2) that this connection does not apply to women, since, even if they have the necessary knowledge, they still fall victim to impulse buying. Again, further studies are needed to shed light on these results.
– (Self-reported) knowledge in finance seems to be an important predictor for budgeting, at least for females. It is interesting to note that this does not apply to delay of gratification or to the control facet. However, one can assume that spontaneous purchases are made against women’s better knowledge.
– It is also interesting to note that a migration background seems to be more relevant for women than for men. Women with an immigrant background have less ability to delay gratification than women without an immigrant background. These differences are not found for men. Why this is the case can be explained neither on the basis of previous studies nor with our data. Again, further studies are needed.
– Delay of gratification has a positive effect on both facets of financial literacy for both male and female participants. This underscores the importance of this variable.

Educational background as a moderator:

– A significant moderating effect of the educational background (academic or vocational) on the connection between self-reported financial literacy and delay of gratification can be determined. While there is a negative relationship between these two variables for people with an academic educational background, it is positive for people with a vocational educational background. One explanation could be that people with an academic background rate their knowledge (too) highly and feel that they have their financial situation under control. Delay of gratification may therefore not be considered necessary.

These results make it clear that it was worthwhile to examine the relationships between the individual characteristic, delay of gratification and financial literacy beyond the hypotheses. Generally, the results show that, in terms of financial literacy, we are dealing with a complex interaction of variables that influence behavior and, in the long-term, financial well-being. The first and most important step is to assess the competence in financial literacy of a given target group and then to develop an adequate and individualized support measure against the background of different socio-demographic factors and personality traits.

Despite the promising results, the study has some limitations. In line with many other SJTs, the reliability of the scales in terms of internal consistency, as measured with Cronbach’s alpha, is rather moderate. Catano et al. (2012) present results from a meta-analysis and report an average internal consistency of 0.46. They explain this relatively low outcome with the fact that even people who obtain a similar result in a construct may act differently in concrete situations. Moreover, an SJT represents a behavior-based simulation of a criterion behavior that is not a “pure” construct (Muck, 2013). Since many constructs require multiple skills, the search for unidimensional factors can be limiting. To act reasonably in financial decision situations, people need many dispositions, including financial knowledge, mathematical knowledge, and the ability to delay gratification. According to Catano et al. (2012), applying reliability estimates other than internal consistency (e.g., retest reliability from a longitudinal perspective) may provide additional insights in this regard.

Furthermore, a more differentiated consideration of the migration background would be helpful. Here, taking into account, the language spoken in the parental home seems to be particularly promising (see Happ et al., 2018). In addition, there is a certain risk that the assessment of delay of gratification conducted *via* the questionnaire is not sufficiently discriminating from the assessment of financial literacy performed with the SJT. Therefore, there might be a certain amount of confounding. Furthermore, only a few factors influencing delay of gratification and financial literacy could be identified, so the explanation of variance is not particularly high, especially in the case of delay of gratification (8%). Here, it would seem to be helpful to include and investigate other influencing factors. In particular, the educational background of the parents or detailed information on the use of informal opportunities to learn seems to be promising. A further limitation is the rather small sample.

## Data Availablity Statement

The raw data supporting the conclusions of this article will be made available by the authors, without undue reservation.

## Ethics Statement

Ethical review and approval was not required for the study on human participants in accordance with the local legislation and institutional requirements. The patients/participants provided their written informed consent to participate in this study.

## Author Contributions

CS and EW contributed to the conception and the design of the work. CS did the analysis and interpretation of the data. CS and EW wrote the manuscript. CS and EW agreed all aspects of the work and approved the final version of the article to be published. All authors contributed to the article and approved the submitted version.

## Conflict of Interest

The authors declare that the research was conducted in the absence of any commercial or financial relationships that could be construed as a potential conflict of interest.

## Publisher’s Note

All claims expressed in this article are solely those of the authors and do not necessarily represent those of their affiliated organizations, or those of the publisher, the editors and the reviewers. Any product that may be evaluated in this article, or claim that may be made by its manufacturer, is not guaranteed or endorsed by the publisher.

## Footnotes

^1^With the term “non-cognitive,” we want to contrast conceptions of financial literacy that solely consider cognitive aspects, such as knowledge, abilities, and skills. Of course, we are aware that this distinction is of an analytical nature and that non-cognitive aspects, such as motivation and attitudes, also contain cognitive elements.

^2^Even though a recent study by Watts et al. (2018) replicates these results only partly and points to the fact that associations between early delay abilities of children and later achievement are highly sensitive to the inclusion of controls and are not stable, in the domain of financial literacy, it can nevertheless be expected that saving and spending behavior are influenced by this ability.
